# Pityriasis Lichenoides Chronica: A Diagnostic and Therapeutic Challenge

**DOI:** 10.7759/cureus.117187

**Published:** 2026-09-29

**Authors:** Kiran Kumre, Binod Kumar, Madhavi Thombare, Ratan Kumar, Anil Prasad

**Affiliations:** 1 Dermatology, Tata Main Hospital, Jamshedpur, IND; 2 Pediatrics, Manipal Tata Medical College, Manipal Academy of Higher Education (MAHE), Jamshedpur, IND; 3 Pediatrics, Tata Main Hospital, Jamshedpur, IND; 4 Pathology and Laboratory Medicine, Tata Main Hospital, Jamshedpur, IND

**Keywords:** a case report, mica, pityriasis lichenoides chronica, s: methotrexate, ulceronecrotic

## Abstract

Pityriasis lichenoides (PL) is an uncommon, acquired inflammatory skin disorder that presents considerable diagnostic and therapeutic challenges. Its classification, natural history, and optimal management remain subjects of debate. PL is best conceptualized as a clinical spectrum encompassing pityriasis lichenoides et varioliformis acuta (PLEVA), pityriasis lichenoides chronica (PLC), and the rare febrile ulceronecrotic Mucha-Habermann disease (FUMHD). Whether these represent truly distinct entities or points along a single continuum remains controversial, given their overlapping clinical, histopathological, and etiological features. PLC typically manifests as an insidious eruption of small, red-to-brown, flat-topped papules bearing a characteristic adherent mica-like scale, following a relapsing-remitting course with prolonged periods of quiescence. We report a case of longstanding PLC in a 60-year-old woman who was successfully managed with a combination of methotrexate, topical corticosteroids, keratolytics, and emollients, achieving sustained remission at six-month follow-up.

## Introduction

Pityriasis lichenoides (PL) is a papulosquamous disorder of uncertain etiology that presents along a spectrum ranging from acute to chronic forms [1]. Pityriasis lichenoides chronica (PLC) represents the milder, more indolent end of this spectrum, whereas pityriasis lichenoides et varioliformis acuta (PLEVA) constitutes the acute variant [2]. Although the precise cause of PL remains unknown, an aberrant T-cell-mediated immune response triggered by an infectious agent has been proposed as the underlying mechanism, and antibodies against *Toxoplasma gondii*, cytomegalovirus, parvovirus B19, adenovirus, and Epstein-Barr virus have been reported in a subset of patients [3]. Definitive diagnosis relies on the correlation of clinical morphology with histopathological findings. We describe an illustrative case of PLC in an elderly woman, highlighting the diagnostic considerations and therapeutic approach relevant to this often under-recognized condition.

## Case presentation

A 60-year-old woman presented to the dermatology outpatient department with a 15-year history of multiple scaly, mildly elevated lesions distributed over the trunk and both upper and lower limbs. The lesions were asymptomatic and had evolved gradually to involve an extensive body surface area. She had not received any prior treatment and denied any associated systemic illness.

Cutaneous examination revealed numerous small, flat-topped, reddish-brown papules bearing fine, centrally adherent, mica-like scales that were readily detachable. The lesions were ovoid and ranged from 5 to 15 mm in diameter. Several older plaques had flattened and resolved spontaneously, leaving transient hypopigmented or hyperpigmented macules in their wake (Figure 1).

**Figure 1:**
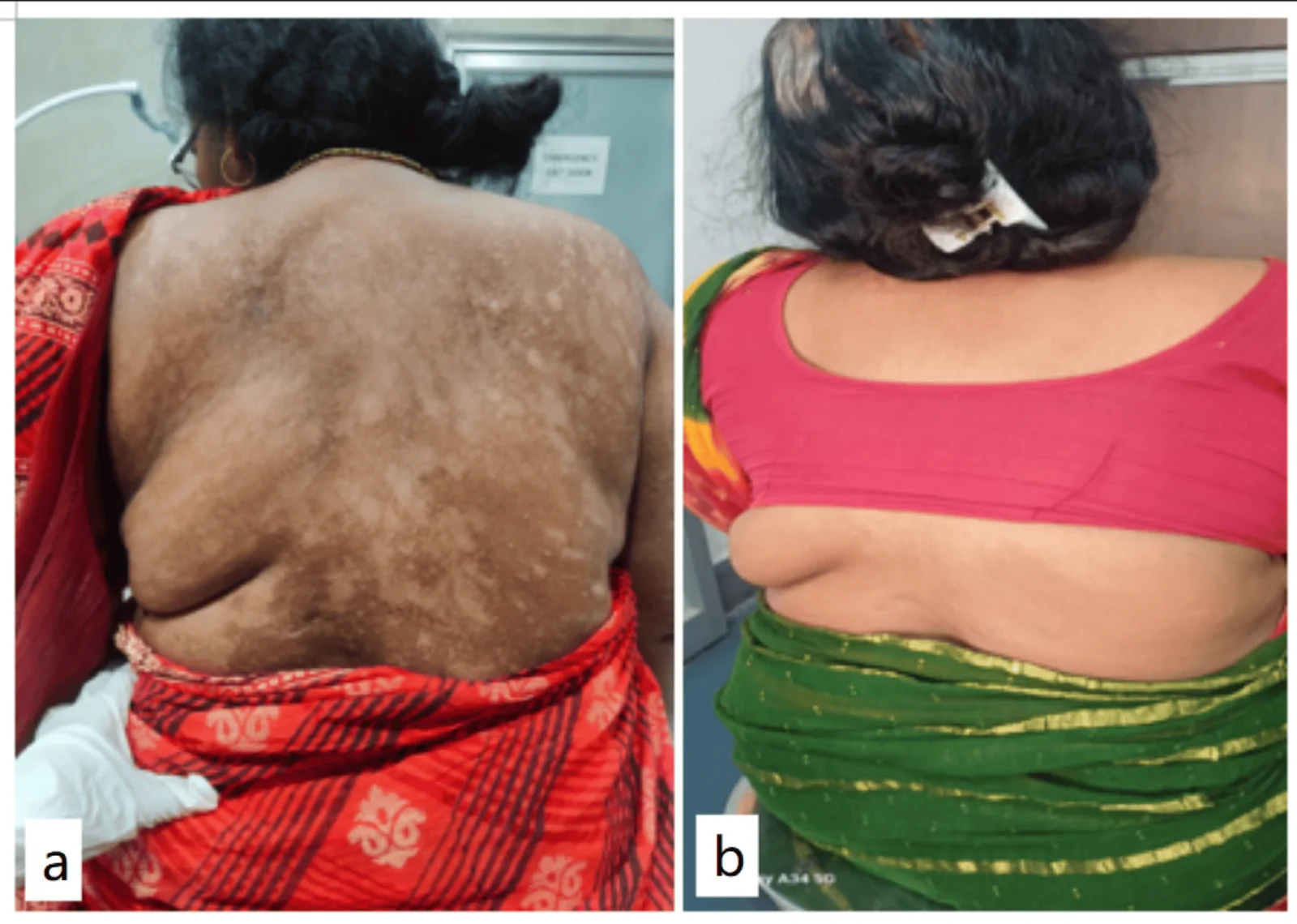
Clinical Image of lesions (a) before treatment, (b) after treatment

Baseline investigations, including complete blood count, urinalysis, liver and renal function tests, Mantoux test, and chest radiograph, were unremarkable. After obtaining informed consent, a punch skin biopsy was performed, which confirmed the diagnosis of pityriasis lichenoides chronica (Figure 2). Amoxyclav was prescribed for three days after the skin biopsy to prevent secondary bacterial infection at the biopsy site.

**Figure 2:**
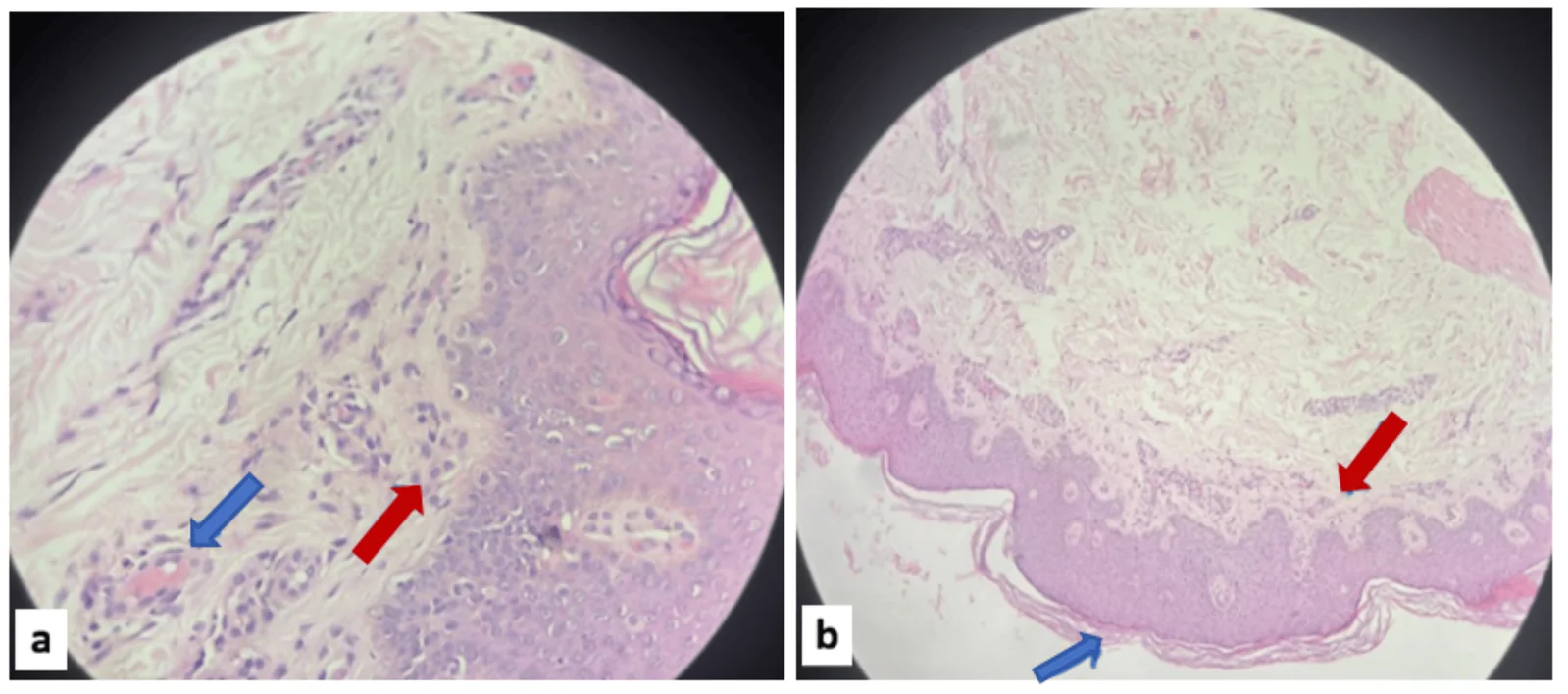
Histopathological examination of the sample from the skin lesion. (a) High magnification (400×): Perivascular and lichenoid lymphocytic infiltrate (blue arrow); extravasated erythrocytes (red arrow). (b) Low magnification (100×): Parakeratosis (blue arrow), Acanthosis, spongiosis, minimal vacuolar degeneration of the basal layer with exocytosis of small numbers of lymphocytes (red arrow). (Stain used: hematoxylin and eosin).

The patient was treated with a combination of topical corticosteroids, keratolytic agents, and oral methotrexate for three months. Pre-treatment assessment of hepatitis B and C status was confirmed to be negative. Methotrexate dose given was 5 mg/week initially, and adverse effects were closely monitored. Complete blood count, liver function test and kidney function tests were done routinely. With good tolerance and no adverse effects, the dose of methotrexate was increased to 7.5 mg/week. This regimen resulted in a marked improvement of the lesions, with no recurrence noted over six months of subsequent follow-up.

## Discussion

PLC is an uncommon, chronic dermatosis of unclear etiology, with an estimated prevalence of up to 0.5% [4]. It is characterized by recurrent crops of small, erythematous-to-hyperpigmented, scaly papules bearing a distinctive fine, adherent, mica-like scale, most commonly affecting the trunk and proximal extremities, and typically asymptomatic or only mildly pruritic.

The pathophysiology of PL remains incompletely understood. The proposed mechanisms include a clonal T-cell lymphoproliferative process arising from an aberrant immune response to an infectious trigger, hypersensitivity-type immune reactions, and associations with viral pathogens, particularly Epstein-Barr virus and cytomegalovirus [3,4]. Some authors have also raised the possibility of PLC representing a T-cell dyscrasia with a small but recognized potential for evolution toward lymphomatoid papulosis or, rarely, cutaneous T-cell lymphoma, underscoring the importance of long-term follow-up [5].

Histopathological examination remains central to confirming the diagnosis. Characteristic features include mild acanthosis, focal parakeratosis, hypogranulosis, focal epidermal necrosis or keratinocyte apoptosis, lymphocytic exocytosis, a superficial and deep perivascular lymphocytic infiltrate, interface dermatitis, and occasional extravasated erythrocytes. The infiltrate is predominantly composed of CD8-positive T cells, although the immunophenotype can vary among patients [5,6].

The differential diagnosis of PLC is broad and includes guttate psoriasis, pityriasis rosea, secondary syphilis, lichen planus, lymphomatoid papulosis, papular urticaria, drug-induced lichenoid eruption, superficial fungal infection, and hypopigmented mycosis fungoides. Careful clinicopathological correlation is essential to exclude these mimickers, particularly lymphoproliferative disorders [6].

The management of PLC remains largely empirical, and most available therapies provide only partial or transient benefit. First-line options include topical corticosteroids, narrowband ultraviolet B (UVB) phototherapy, and tetracyclines such as doxycycline or minocycline for their anti-inflammatory properties [7]. In refractory or extensive disease, topical calcineurin inhibitors, methotrexate, cyclosporine, and psoralen plus ultraviolet A (PUVA) phototherapy have been used with variable success [4,7,8]. In our case, in view of the extensive and long-standing disease course as well as non-availability of phototherapy unit/narrow band UVB phototherapy option at our center, methothrexate was chosen as the therapeutic option after a discussion with the family members. A systematic review of treatment outcomes in pityriasis lichenoides found that phototherapy achieved the highest response rates, followed by topical corticosteroids and antibiotics, while noting an overall paucity of high-quality comparative trials to guide therapy [9]. Emerging biologic approaches, including dupilumab, have also been reported anecdotally in recalcitrant cases, reflecting an ongoing interest in targeted immunomodulation for this disease [10]. In our patient, a combination of methotrexate with topical corticosteroids, keratolytics, and emollients achieved sustained clearance, consistent with reports supporting methotrexate as an effective option in long-standing, treatment-resistant PLC. Post-inflammatory pigmentary changes, whether hypo- or hyperpigmented, often persist considerably longer than the active papulosquamous lesions themselves and should be discussed with patients to set realistic expectations regarding cosmetic recovery.

We acknowledge the limitations of our study being a single case report and lack of a controlled comparative group. Moreover, the observed response should be interpreted as a clinical observation that may warrant long-term follow-up.

## Conclusions

Pityriasis lichenoides chronica is a chronic, relapsing papulosquamous disorder that requires careful distinction from other benign, lymphoproliferative, and malignant mimickers. Skin biopsy remains essential for confirming the diagnosis and excluding more serious conditions. Although PLC is generally benign, its course can extend over months to years, and treatment is aimed primarily at symptom control and acceleration of lesion resolution. Our case illustrates that methotrexate, in combination with topical therapies, can achieve durable remission in longstanding, treatment-refractory disease, supporting its consideration in patients who fail conventional first-line measures.
